# Improving family communication after a new genetic diagnosis: a randomised controlled trial of a genetic counselling intervention

**DOI:** 10.1186/1471-2350-15-33

**Published:** 2014-03-14

**Authors:** Jan M Hodgson, Sylvia A Metcalfe, MaryAnne Aitken, Susan M Donath, Clara L Gaff, Ingrid M Winship, Martin B Delatycki, Loane LC Skene, Belinda J McClaren, Jean L Paul, Jane L Halliday

**Affiliations:** 1 Department of Paediatrics, University of Melbourne, Melbourne, Australia; 2 Murdoch Childrens Research Institute, MCRI, 5th Floor Royal Childrens Hospital, Melbourne 3052, Australia; 3 Walter and Eliza Hall Institute, Melbourne, Australia; 4 Genetic Medicine, Royal Melbourne Hospital, Melbourne, Australia; 5 Department of Medicine, University of Melbourne, Melbourne, Australia; 6 Bruce LeFroy Centre for Genetic Health Research, MCRI, Melbourne, Australia; 7 Department of Genetics, Austin Health, Melbourne, Australia; 8 Melbourne Law School, University of Melbourne, Melbourne, Australia

**Keywords:** Genetic counselling, Family communication, Genetic diagnosis, Disclosure

## Abstract

**Background:**

Genetic information given to an individual newly diagnosed with a genetic condition is likely to have important health implications for other family members. The task of communicating with these relatives commonly falls to the newly diagnosed person. Talking to relatives about genetic information can be challenging and is influenced by many factors including family dynamics. Research shows that many relatives remain unaware of relevant genetic information and the possible impact on their own health. This study aims to evaluate whether a specific genetic counselling intervention for people newly diagnosed with a genetic condition, implemented over the telephone on a number of occasions, could increase the number of at-risk relatives who make contact with genetics services after a new genetic diagnosis within a family.

**Methods:**

This is a prospective, multi-centre randomised controlled trial being conducted at genetics clinics at five public hospitals in Victoria, Australia. A complex genetic counselling intervention has been developed specifically for this trial. Probands (the first person in a family to present with a diagnosis of a genetic condition) are being recruited and randomised into one of two arms – the telephone genetic counselling intervention arm and the control arm receiving usual care. The number of at-risk relatives for each proband will be estimated from a family pedigree collected at the time of diagnosis. The primary outcome will be measured by comparing the proportion of at-risk relatives in each arm of the trial who make subsequent contact with genetics services.

**Discussion:**

This study, the first randomised controlled trial of a complex genetic counselling intervention to enhance family communication, will provide evidence about how best to assist probands to communicate important new genetic information to their at-risk relatives. This will inform genetic counselling practice in the context of future genomic testing.

**Trial registration:**

Australia and New Zealand Clinical Trials Register (ANZCTR): ANZCTRN12608000642381.

## Background

When an individual (proband) receives a new diagnosis of a genetic condition for themselves or their child, the genetic information often has implications for other family members who may themselves be at risk of carrying the same mutation. This means that they may develop the condition themselves or risk passing the mutation on to their children.

Current local practice in state funded health services in Victoria, Australia, in keeping with Victorian Privacy laws, is for genetic health care providers to discuss with the proband the importance of communicating the genetic information to at-risk relatives and offer resources such as letters or information sheets. The health care provider, however, does not make direct contact with relatives. Despite routine advice given to the proband about the importance of communicating risk to relatives, it is estimated that between 20 and 40% of at-risk relatives remain unaware of the relevant genetic information [1-3]. Several reasons have been identified for this failure of family communication. First, genetic information is often difficult to understand and to explain clearly, and probands may lack knowledge or confidence in their ability to communicate this information [4-6]. Further, while probands frequently describe feeling a sense of obligation about informing other at-risk relatives [7], they may be reluctant to do so due to existing challenging family dynamics and relationships [8,9]. Finally, probands may wish to protect their family members from anxiety [10-13] or they may be experiencing difficulties in coming to terms with their own diagnosis, [1,14] making communication problematic.

Most knowledge of how families communicate genetic information comes from research into families with inherited cancers. It is recognised that probands frequently deliberate about when may be an appropriate time for their relatives to receive the genetic information [15]. Women are more likely to communicate than men [16] and, when communication occurs, it appears that first-degree relatives are usually informed first and often with an assumption that those who are informed will in turn pass the information on to their close relatives [1].

When questioned about how communication of genetic information should optimally occur, relatives believe that it should be done by family members but supported by genetic health professionals [12]. Health professionals themselves have often debated whether they should take a more active approach in informing possibly at-risk family members about genetic information. Professional guidelines encourage discussion of the importance of family communication [17] and there have been some attempts by genetics services to assist probands to do so by asking for contact details and sending letters to at-risk relatives on their behalf [18] or by use of a genetic register [19].

Overall, it appears that probands require more assistance to communicate genetic information effectively [6,20]. A small study in Australia provided evidence that additional genetic counselling support could result in a significant increase (36-61%) in family communication, represented by an increase in contact from at-risk family members with genetics services [19].

## Presentation of the hypothesis

The aim of this multicentre randomised trial is to investigate whether a specifically designed genetic counselling intervention will result in increased access to genetics services by at-risk family members, following identification of a genetic condition or carrier status in a proband.

The main hypothesis is that a specifically designed genetic counselling intervention delivered by phone at three time points over 18 months will facilitate communication of important genetic information within families, thereby increasing the number of at-risk family members who seek information and/or testing from genetics services.

## Testing the hypothesis

The following details are presented in accordance with the CONSORT reporting guidelines for randomised trials of non-pharmacologic treatment [21]. The following Human Research Ethics Committees in Victoria, Australia have all approved this project: Austin Health, Bendigo Health, Ballarat Health, Royal Childrens Hospital, Melbourne Health and Southern Health.

### Participants

#### Inclusion criteria

Potential participants for this prospective, randomised controlled trial must:

• be the first person in a family to be diagnosed, or have a child diagnosed, with a genetic condition about which the genetic information has implications for other family members and for which genetic testing or diagnosis based on clinical information is possible

• be able to speak, read and write English

• have at least one at-risk relative who resides in Victoria

• be aged 18 years or over.

#### Exclusion criteria

Potential participants are excluded if they:

• have a cognitive disorder that affects their ability to give informed consent

• have received a diagnosis during pregnancy

• reside outside of Victoria.

#### Setting and locations

The trial is being conducted within genetic clinic settings at six tertiary public hospitals in Victoria. These sites were chosen on the basis of variation in client populations and types of genetic diagnoses likely to be made, as well as the presence of an experienced genetic counsellor on staff.

#### Genetic counsellors performing the intervention

The 10 genetic counsellors in the study routinely disclose new genetic information to probands as part of their clinical duties. Each genetic counsellor is either fully certified or has a minimum of two years clinical experience. All counsellors have undertaken specific training in delivering the genetic counselling intervention and have access to an intervention protocol manual.

### Study intervention

Development of the intervention was informed by a framework for complex interventions [22]. This enabled the intervention to incorporate the patient-centred ethos and nondirective stance fundamental to genetic counselling practice with a process that is standardised and can be replicated. The intervention was designed to use genetic counselling strategies to address documented personal barriers to family communication. The Reciprocal Engagement Model [23] is the model of genetic counselling practice applied in the intervention, while evidence for barriers was taken from a systematic literature review [1].

The counselling framework for the intervention has three domains; the focus on each is determined by the participant’s needs over time. These are:

• “Getting the picture” about the participant’s experiences of communication to date, including: which relatives have been informed and their responses; recognising and exploring the conscious and unconscious barriers to communication; supporting adjustment of the participant to their own genetic status.

• Forming an intention to act. This includes maintaining or enhancing the participant’s capacity for communication; facilitating decision-making; and resolving ambivalence.

• Planning to act. Here the participant intends to communicate, options are elicited, a plan developed and potential scenarios explored to prepare the participant.

The intervention was designed to be feasible to implement in clinical practice, as an adjunct to usual care [24].

To further emulate the ‘real world setting’ the intervention is delivered by the same genetic counsellor that each participant met at their visit to the genetics clinic.

Intervention phone calls are audiotaped and analysed to examine adherence to the intervention protocol. Such monitoring of the intervention fidelity ensures its delivery and receipt is as intended.

### Process

At the time of their initial genetic consultation all probands receive standard care concerning family communication from the genetic counsellor. This generally involves a discussion of possible implications for other family members and the offer of explanatory letters for the proband to pass on to at-risk relatives Probands are informed about the research study by the genetic counsellor at the end of this consultation. If they consent, their contact details are faxed to the research team for recruitment to proceed. A researcher then contacts potential participants by telephone to explain the project and the possibility that they may receive follow-up telephone calls (if in the intervention arm). Written informed consent is requested by mail and, once received, participants are randomised into the intervention or control group.

Participants who are randomised into the control group receive no further contact specific to family communication.

Participants in the intervention group receive standard care plus three intervention phone calls from a genetic counsellor delivered over the subsequent 12 months.

### Outcomes

#### Primary – measurement of uptake of genetic services

The number of at-risk relatives is ascertained from three generation pedigrees collected by the genetic counsellors at the time of diagnosis for all participants and held in the family Genetics Files. Eighteen months after the proband attends the genetic clinic and is enrolled in the study, the family file is audited to determine the actual number of at-risk relatives who make contact with Victorian genetic clinics.

The total number of at-risk relatives who have contact with a genetics service divided by the total number of at-risk relatives gives a percent that can be compared between the two arms of the study. A person’s ‘at-risk status’ is based on genetic relatedness, taking into account the inheritance pattern for the condition involved. During the study there may be further clarification of the at-risk status. For example, a negative genetic test result for an autosomal recessive condition in one family member means that the mutation will not be further transmitted in that branch of the family. Therefore, the original estimate of the number of ‘at-risk’ relatives will be reduced accordingly. Relatives who live overseas or interstate are excluded from the analysis as we are unable to document their uptake of genetics services.

All data are managed in ACCESS database, 2007 version.

#### Secondary – follow-up telephone interview to assess participant experiences

At the 18 month exit point of the study, participants are telephoned to complete a structured survey assessing their experiences of family communication and participation in a RCT. This identifies differences in attitudes, awareness and suggestions for best practice between the two groups. This survey has been developed using a Delphi process involving the study team and both experienced and student genetic counsellors. It focuses on:

• awareness of the importance of information for at-risk family members

• experience of informing family members

• which family members were informed and when

• the barriers and facilitators to communication

• professional support that is helpful and acceptable to families

• other suggestions for practice.

In addition, the genetic counsellors who delivered the intervention will be interviewed in a focus group setting to reflect on their experience of participation.

A further sub-study to assess fidelity will examine the intervention phone calls to determine counsellors’ adherence to the intervention protocol.

### Sample size

We aim to detect a difference of at least 15% in contact with a genetic service (e.g. 20% of relatives in the control group, 35% in the intervention group). A sample size of 151 at-risk relatives per group has 80% power to detect this difference (with an alpha level of 0.05) if all the at-risk relatives were independent of each other; that is, if the clustering of at-risk relatives is ignored. Data from a preliminary study in Tasmania suggest that there is an average of seven at-risk relatives per proband. With a conservative estimate of five at-risk relatives per proband, a sample size of 151 at-risk relatives requires participation of 30 probands [19].

Since is it probable that responses of at-risk relatives of each proband are more similar to each other than to relatives of other probands, the clustering effect must be taken into account. The average degree of similarity to be expected between at-risk relatives of a given proband (the intraclass correlation (ICC) is unknown as there have been no similar studies. Data from community-based surveys in the UK have shown that many ICCs for households were in the range of 0.0 to 0.3 [25]. Assuming an ICC of 0.25 and an average cluster size of 5, the Design Effect (DE) would be 2 (DE = 1+ (cluster size -1)*ICC). Thus, the required number of at-risk relatives in each group is 302 (=151*2) necessitating a sample size of 60 probands in each arm. Given our conservative assumption of five at-risk relatives per proband, this will detect a difference of at least 15%. In addition, with attrition rate of approximately 15% over the 18 month study period, there is a need to recruit an extra 10 probands for each arm (i.e. 70 probands per arm).

To allow for correlation between the likelihood of making contact with a genetics service of relatives of the same probands, the method of marginal logistic regression models fitted using Generalised Estimating Equations (GEEs) with information sandwich estimates of standard error will be used to estimate the odds ratios between the intervention and control arm for the primary outcome.

### Randomisation

The randomisation sequence was constructed by the Clinical Epidemiology and Biostatistics Unit (CEBU) at the Murdoch Childrens Research Institute using Stata 11.0 (StataCorp, College Station, TX) statistical software. A statistician in CEBU, who is independent of the study investigators, retains the only copy of the randomisation sequence, Randomisation is stratified by genetic counsellor, allocation is 1:1 and random block sizes are used to ensure concealment of allocation. After a participant is enrolled in the study, the study co-ordinator contacts CEBU to obtain the treatment allocation for that participant.

## Implication of the hypothesis

Successful family communication about important genetic information is dependent on a number of factors including pre-existing family dynamics and an individual’s ability to give and receive complex genetic information. This trial, of a genetic counselling intervention that has been specifically designed to address potential barriers to family communication, will provide evidence for whether additional support by health professionals can improve family communication about genetics.

A project steering committee meets monthly to oversee the trial and ensure that it is being conducted rigorously and in accordance with HREC requirements.

As recruitment for the trial is slower than had been anticipated, a data collection form has been used for a three month period to determine the eligibility of clients being seen at each genetics clinic. This has enabled documentation of reasons for non-participation and those findings will be published separately.

Ascertaining whether family communication actually takes place is problematic as it would be unethical and not feasible to ask at-risk relatives directly whether they have been informed of the presence of a genetic condition in the family. Therefore, it is important to note that the primary outcome measure, the percent of family members who actually make contact with Victorian genetics services, is a ‘proxy’ measure and may not reflect the true level of family communication that in fact occurs.

Another possible reason that the primary outcome might produce an under-representation of family communication is that relatives may access genetic information from agencies, individuals outside the scope of this study, e.g. GPs, obstetricians or non-Victorian genetic services. Given the randomised study design, any such under-enumeration of the primary outcome measure would be expected to be similar in both arms of the study.

Greater understanding of the genetic basis of disease arising from initiatives such as the Human Genome Project combined with technological advances mean that more people will receive definitive genetic diagnoses and testing results. There is also a growing access to, and demand for, genomic information originating outside the clinic setting (e.g. direct-to-consumer testing), but leading to a need for genetic counselling [26]. Consequently, the need for communication of this information within families will become even more prevalent. We anticipate that the findings of this study will assist in development of new resources and enhance skills of health professionals who are required to assist in this process of communication of genetic information in families.

## Competing interests

The authors declare that they have no competing interests.

## Authors’ contributions

JMH wrote the first draft of this manuscript, co-developed the intervention, coordinates the study, participated in design and edited all manuscript drafts. SAM participated in design and coordination of the study, assisted with developing the outcome measures, contributed to manuscript preparation and edited manuscript drafts. MAA conceived of the study, participated in its design and management and edited manuscript drafts. SMD participated in design and management of the study, assisted in developing all analyses and edited manuscript drafts. MBD, LLCS and IMW contributed to design and management of the study and edited manuscript drafts. CLG participated in design and management of the study, co-developed the intervention and edited manuscript drafts. BJM contributed to data collection, design of the outcome measures and edited manuscript drafts. JLP assisted with analysis of the intervention and edited manuscript drafts. JLH (Chief Investigator) conceived of the study, participated in its design, coordination and management, contributed significantly to manuscript preparation and edited manuscript drafts. All authors read and approved the final manuscript.

## Pre-publication history

The pre-publication history for this paper can be accessed here:

http://www.biomedcentral.com/1471-2350/15/33/prepub
